# Differentiated mouse kidney tubuloids as a novel *in vitro* model to study collecting duct physiology

**DOI:** 10.3389/fcell.2023.1086823

**Published:** 2023-01-25

**Authors:** C. J. A. Olde Hanhof, E. Dilmen, F. A. Yousef Yengej, F. Latta, C. M. E. Ammerlaan, J. Schreurs, L. Hooijmaijers, J. Jansen, M. B. Rookmaaker, I. Orhon, M. C. Verhaar, J. G. Hoenderop

**Affiliations:** ^1^ Department of Molecular Physiology, Radboud Institute for Molecular Life Sciences, Radboud University Medical Center, Nijmegen, Netherlands; ^2^ Hubrecht Institute, Royal Netherlands Academy of Arts and Sciences, Utrecht, Netherlands; ^3^ Department of Nephrology and Hypertension, University Medical Center Utrecht, Utrecht, Netherlands; ^4^ Department of Pathology, Radboud Institute for Molecular Life Sciences, Radboud University Medical Center, Nijmegen, Netherlands; ^5^ Department of Pediatric Nephrology, Radboud Institute for Molecular Life Sciences, Radboud University Medical Center, Amalia Children’s Hospital, Nijmegen, Netherlands; ^6^ Institute of Experimental Medicine and Systems Biology, Medical Faculty RWTH Aachen University, Aachen, Germany

**Keywords:** tubuloid, organoid, epithelial sodium transport, cell physiology, collecting duct, tubulopathy

## Abstract

Kidney tubuloids are cell models that are derived from human or mouse renal epithelial cells and show high similarities with their *in vivo* counterparts. Tubuloids grow polarized in 3D, allow for long-term expansion, and represent multiple segments of the nephron, as shown by their gene expression pattern. In addition, human tubuloids form tight, functional barriers and have been succesfully used for drug testing. Our knowledge of mouse tubuloids, on the other hand, is only minimal. In this study, we further characterized mouse tubuloids and differentiated them towards the collecting duct, which led to a significant upregulation of collecting duct-specific mRNAs of genes and protein expression, including the water channel AQP2 and the sodium channel ENaC. Differentiation resulted in polarized expression of collecting duct water channels AQP2 and AQP3. Also, a physiological response to desmopressin and forskolin stimulation by translocation of AQP2 to the apical membrane was demonstrated. Furthermore, amiloride-sensitive ENaC-mediated sodium uptake was shown in differentiated tubuloids using radioactive tracer sodium. This study demonstrates that mouse tubuloids can be differentiated towards the collecting duct and exhibit collecting duct-specific function. This illustrates the potential use of mouse kidney tubuloids as novel *in vitro* models to study (patho)physiology of kidney diseases.

## Introduction

The kidneys maintain homeostasis of fluids and electrolytes, remove waste products from the blood and regulate blood acid-base balance (Alpern and Hebert, 2007). Kidneys are composed of multiple nephron segments: the proximal tubule (PT), the loop of Henle (LoH), distal convoluted tubule (DCT), connecting tubule (CNT) and finally the most distal part of the nephron, the collecting duct (CD) (Alpern and Hebert, 2007). The principal cells (PCs) of the CD perform final water reabsorption, whereas the intercalated cells (ICs) of the CD are important for maintaining blood acid-base homeostasis (Fushimi et al., 1993; Brown, 2003). PC water reabsorption is promoted by the hormone arginine vasopressin (AVP) (Fushimi et al., 1993; van Lieburg et al., 1995; Brown, 2003). AVP binds to the vasopressin type-2 receptor (V2R), which promotes translocation of the water channel aquaporin 2 (AQP2) towards the apical membrane (Yamamoto et al., 1995; Sandoval et al., 2013; Jung and Kwon, 2019). Basolateral water extrusion is mediated by the AQP3 and AQP4 water channels (Ishibashi et al., 1994; Terris et al., 1995). PCs of the CD also mediate final kidney sodium (Na^+^) reabsorption by the apically expressed amiloride-sensitive epithelial sodium channel (ENaC), a heteromultimeric channel that consists of three subunits, alpha, beta and gamma (Canessa et al., 1994; Bhalla and Hallows, 2008). Basolateral Na^+^ excretion is mediated by the Na^+^/potassium (K^+^)-ATPase (Doucet, 1988). Dysregulation of this CD electrolyte handling can lead to hereditary (e.g., nephrogenic diabetes insipidus, Liddle’s syndrome and pseudohypoaldosteronism type 1a) or acquired diseases that show wasting of electrolytes and/or water (Geller et al., 2006; Enslow et al., 2019; Kavanagh and Uy, 2019; Downie et al., 2021).

Our current understanding of kidney (patho)physiology stems from detailed studies using (immortalized) cell models and/or animal models. It is evident that these cell models are often too simplistic to mimic the complex *in vivo* situation (Jensen and Teng, 2020). A major limitation of immortalized cell lines is their lack of a physiological expression profile of key kidney markers, since conventional immortalization induces cellular dedifferentiation (Jenkinson et al., 2012; Gartzke and Fricker, 2014; Ramboer et al., 2014; Van der Hauwaert et al., 2014; Labarca et al., 2015; Slusser et al., 2015; Yu et al., 2018). Primary kidney cells in culture can overcome this problem, but are known to lose expression of relevant proteins within days (Baer et al., 2006; Terryn et al., 2007; Van der Hauwaert et al., 2013). Although animal models such as mice do provide the context of the *in vivo* situation, they are often time consuming and expensive (Russell and Burch, 1959). Therefore, novel research models are needed to advance understanding of kidney (patho)physiology.

Recently, three-dimensional (3D) kidney organoid models have been developed that can be grown either from induced pluripotent stem cells (iPSCs), first described in 2014, or from adult stem/progenitor cells (ASPCs) as described by Schutgens et al. (2019) Clevers (2016). The directed differentiation of iPSC-derived organoids recapitulates nephrogenesis and produces multiple cell types that have similar maturity to the first or second trimester of the human fetal kidney (Takasato et al., 2015; Wu et al., 2018; Combes et al., 2019). ASPC-derived kidney organoids or tubuloids correspond with a more mature expression profile compared to iPSC-derived kidney organoids. Tubuloids are grown in a 3D environment from primary renal epithelial cells by inducing a dedifferentiated progenitor state through amplification of Wnt signaling and activation of receptor tyrosine kinases. ASPC-derived kidney organoids consist solely of polarized adult kidney tubular epithelium and are, therefore, also referred to as tubuloids (Schutgens et al., 2019). Schutgens et al. (2019) showed that tubuloids could be obtained from human and mouse primary kidney tissue and could be maintained in culture for at least 20 weekly passages while retaining genetic stability. Human tubuloids were shown to express markers of different nephron segments; predominantly the PT, limited expression of the LoH, DCT and the CD and absence of glomerular cells. Interestingly, growth factor withdrawal in tubuloids further enhanced expression of markers of the distal part of the nephron. The first proof of principle experiments confirmed that human tubuloids formed tight and intact barriers, could be used in an organ-on-a-chip system, and contained functional transporters (P-glycoprotein) (Schutgens et al., 2019; Gijzen et al., 2021). Also, tubuloids have been used to generate a large biobank of kidney cancers and for nephrotoxicity screening (Calandrini et al., 2020; Schutgens et al., 2021; Wiraja et al., 2021). However, in contrast to the detailed reports of human tubuloids, our knowledge of mouse tubuloids is limited (Schutgens et al., 2019).

Here, we further characterize the mouse kidney tubuloid model and describe a protocol to differentiate tubuloids towards the CD. We then validate the mRNA and protein expression of multiple CD transporters/channels and assess functionality of the tubuloid model. A functional *in vitro* CD mouse model allows for detailed studies of kidney (patho)physiology and complements existing mouse models of, e.g., CD tubulopathies. In this study, mouse tubuloid CD differentiation resulted in significantly upregulated CD mRNA and proteins. Functional studies with CD-enriched tubuloids demonstrated amiloride-sensitive ENaC-mediated Na^+^ uptake. Therefore, mouse kidney tubuloids are proposed as a novel model to study kidney CD physiology *in vitro*.

## Materials and methods

### Animals

Wild-type female C57BL/6 mice (*Mus musculus*) were kept at the animal facility of the Radboud University in Nijmegen. Animals were housed with six per cage under standard conditions with bedding in a temperature-controlled room with a 12-h light/dark cage. Water and standard pellet chow were available *ad libitum* (Ssniff Spezialdiäten, Soest, Germany). Mice were sacrificed at the age of 24 days by cervical dislocation and their kidneys were harvested. The animal procedures were performed in accordance with the guidelines of the Animal Ethics Board of the Radboud University Nijmegen.

### Mouse-derived tubuloid culture

Kidneys from 2 C57BL/6 mice (hereafter referred to as tubuloid line A and B) were digested by 1 μg/mL collagenase (LS004194, Worthington) treatment for 1.5 h to obtain tubular fragments. Fragments were embedded in Cultrex reduced growth factor Basement Membrane Extract (BME) type 2 (3533-001-02, R&D Systems) and cultured in expansion medium (EM) consisting of basal medium (BM) (advanced DMEM/F12 (12634028, Thermo Fisher Scientific) supplemented with 1% (v/v) penicillin/streptomycin, 1% (v/v) HEPES (H3375, Sigma-Aldrich) and 1% (v/v) GlutaMAX (35050038, Thermo Fischer Scientific) with 1.5% (v/v) B-27 supplement (17504044, Gibco), 1% (v/v) RSPO3-Fc fusion protein conditioned medium (R001, U-Protein Express BV), N-acetylcysteine (1 mM, A7250, Sigma-Aldrich), FGF-10 (100 ng/mL, 100-26, Peprotech), A 83-01 (5 μM, SML0788, Sigma-Aldrich), EGF (50 ng/mL, AF-100-15, Peprotech) and Y-27632 (10 μM, HY-10583, MedChem Express) (Table 1). Tubuloids were kept at 37°C with 5% CO_2_ and medium was changed three times per week. Tubuloids were passaged 1:2 to 1:3 weekly by mechanical shearing with a flame-polished pipette (Gijzen et al., 2021). Tubuloid CD differentiation medium (CM) consisted of BM supplemented with forskolin (10 μM, F6886, Merck), A 83-01 (5 μM, SML0788, Merck) and PD0325901 (1 μM, S1036, Pfizer) (Table 1). Tubuloid desmopressin (DDAVP) stimulation medium consisted of BM supplemented with DDAVP (10 nM, V1005, Sigma-Aldrich), A 83-01 (5 μM) and PD0325901 (1 μM) (Table 1).

**TABLE 1:** Composition of the media used.

Medium	Components
Growth factor withdrawal medium (BM)	Advanced DMEM/F12 with penicillin/streptomycin (1% v/v), HEPES (1% v/v), and GlutaMAX (1% v/v)
Expansion medium (EM)	BM with B-27 supplement (1.5% v/v), RSPO3 conditioned medium (1% v/v), N-acetylcysteine (1 mM), FGF-10 (100 ng/mL), A 83-01 (5 µM), EGF (50 ng/mL), and Y-27632 (10 µM)
CD differentiation medium (CM)	BM with forskolin (10 µM), A 83-01 (5 µM), and PD0325901 (1 µM)
Desmopressin (DDAVP) stimulation medium	BM with DDAVP (10 nM), A 83-01 (5 µM), and PD0325901 (1 µM)
CD stimulation medium	BM with forskolin (10 µM), A 83-01 (5 µM), and fludrocortisone acetate (10 µM)

### RNA isolation, cDNA generation and real time quantitative PCR (RT-qPCR)

Total RNA was isolated from tubuloids using the Nucleospin RNA XS kit (740902.50, Macherey-Nagel) according to the manufacturer’s instructions. To obtain cDNA, the RNA was subjected to reverse transcription using M-MLV according to manufacturer’s instructions (28025013, Thermo Fisher Scientific). Subsequently, the diluted cDNA was used to determine gene expression levels using the CF96 real time PCR detection system (185-4095-IVD, Bio-Rad). PCR program was as follows: 1) 7 min 95°C; 2) 15 s 95°C (denaturation); 3) 1 min 60°C; 4) back to 2) for 39 cycles; 5) 10 s 95°C; 6) 5 s/0.5°C 60°C–95°C. All gene expression levels were normalized to *Hprt* housekeeping gene. The primer sequences can be found in Table 2. Relative expression values were determined using the ΔΔCT method, where the control condition was arbitrarily set at 1.

**TABLE 2:** Primers.

Target gene	Forward (5′-3′)	Reverse (5′-3′)
*Hprt*	TTG​CTG​ACC​TGC​TGG​ATT​AC	AGT​TGA​GAG​ATC​ATC​TCC​AC
*Aqp2*	CTT​TGC​CTC​CAC​TGA​TGA​GC	GGAGCGGGCTGGATTCAT
*Aqp3*	TTT​GGA​CCT​CGC​CTC​TTC​AC	TGA​GCT​GGT​ACA​CGA​AGA​CA
*Scnn1a*	CAT​GCC​TGG​AGT​CAA​CAA​TG	CCA​TAA​AAG​CAG​GCT​CAT​CC
*Scnn1g*	TGA​CCT​GCT​TCT​TCG​ATG​GG	TTG​CAG​ACC​ATA​CTC​ACT​GCC
*Avpr2*	CTC​ATC​ATC​AGC​CAC​CAC​AC	GGA​GAG​CTA​GGG​GAC​GAA​AG
*Anpep*	ACC​CCA​ACA​ACC​TCA​TAG​CT	ACT​CAG​TCA​TGG​TGC​AGG​AA
*Umod*	ACT​GCA​CCG​ATC​CTA​GTT​CC	CAC​TCC​AGC​CTG​TAC​TCC​AA
*Pvalb*	GAC​GGC​AAG​ATT​GGG​GTT​G	ACTGAGATGGGGCGTTGG
*Calb1*	GAC​GGA​AGT​GGT​TAC​CTG​GA	ATT​TCC​GGT​GAT​AGC​TCC​AA
*Slc4a1*	TGA​TGT​TTG​CCT​CCG​TTC​TG	AGC​CCT​TGA​TCA​TCT​TCC​GT
*Slc14a2*	GGA​CCT​GAG​TGA​CTG​GCT​ATT​T	ATC​TCC​TTC​AGG​GGG​TGG​TG

### Western blot

Tubuloid samples were lysed with Triton lysis buffer (50 mM Tris-HCl pH 7.5, 1 mM EGTA, 1 mM EDTA, 1% (v/v) Triton X-100 (X100, Sigma-Aldrich), 10 mM sodium glycerophosphate, 1 mM sodium orthovanadate, 50 mM sodium fluoride, 10 mM sodium pyrophosphate, 270 mM sucrose and 150 mM sodium chloride) supplemented with protease inhibitors (1 μg/mL pepstatin (0219536825, MP Biochemicals), 5 μg/mL leupeptin (0215155380, MP Biochemicals), 1 μM phenylmethanesulfonyl fluoride (P7626, Sigma-Aldrich) and 1 μg/mL aprotinin (1371803, Serva Electrophoresis)) and 0.1% (v/v) beta-mercaptoethanol (M6250, Sigma-Aldrich). Subsequently, proteins were denatured in Laemmli sample buffer (2% (v/v) SDS (205-788-1, Serva), 0.01% (w/v) bromophenol blue (161-0404, Bio-Rad), 6% (v/v) glycerol (800688, MP Biochemicals), and 60 mM Tris-HCl/pH 6.8) containing 100 mM of DTT (04856126, MP Biochemicals) for 30 min at 37°C. Protein samples were run on 10% (v/v) (for AQP2) and 8% (v/v) (for ENaC) SDS-PAGE gel and transferred to a methanol-activated polyvinylidene difluoride membrane (IPVH00010, Millipore). The immunoblots were then blocked rotating in 5% (w/v) non-fat dried milk (NFDM) in TBS-T (0.3% (v/v) Tween (0777, VWR Chemicals), 10 mM Tris pH 8, and 150 mM NaCl) for 45 min. Following this, blots were incubated rotating in primary antibodies (Table 3) diluted in 1% (w/v) NFDM in TBS-T overnight at 4°C. Membranes were washed four times in TBS-T, followed by incubation while rolling with secondary HRP-conjugated antibodies diluted in 1% (w/v) NFDM in TBS-T for 1 h at 4°C. The blots were visualised using the ImageQuant LAS 4000 (GE Healthcare) after applying SuperSignal West Pico PLUS Chemiluminescent Substrate (34580, Thermo Fisher Scientific).

**TABLE 3:** Antibodies/stains.

Antibody/stain	Origin	Dilution	Manufacturer
Alexa Fluor 488/594-conjugated antibodies	Goat	IHC 1:300	Thermo Fisher Scientific
AQP2	Guinea Pig	IHC 1:50	Homemade antibody Deen et al. (1994)
		WB 1:1,000	
AQP3	Rabbit	IHC 1:50 + TSA	Homemade antibody van Balkom et al. (2003)
Beta-actin	Mouse	WB 1:10,000	5441, Sigma Aldrich
Biotin-conjugated antibody	Goat	IHC 1:2,000	4050-08, Southern Biotech
DAPI		IHC 12.5 μg/mL	D1306, Invitrogen
E-cadherin	Rat	IHC 1:50	14-3249-82, Invitrogen
Alpha subunit of ENaC	Rabbit	WB 1:1,000	SPC-403D, StressMarq
Gamma subunit of ENaC	Rabbit	WB 1:1,000	Homemade antibody Masilamani et al. (1999)
Na^+^/K^+^-ATPase	Rabbit	IHC 1:200	Kind gift from Prof. Koenderink, Homemade antibody Koenderink et al. (2003)
PO-conjugated anti rabbit	Goat	WB 1:10,000	A4914, Sigma Aldrich
PO-conjugated anti human/rat/rabbit/mouse/guinea pig	Mouse	WB 1:10,000	A5441, Sigma Aldrich
ZO-1	Mouse	IHC 1:50	339100, Invitrogen

### Immunohistochemistry (IHC) of 3D tubuloids

Tubuloids were fixated in 4% (w/v) formalin (4078-9001, KLINIPATH) for 15 min. Next, the tubuloids were collected and embedded in a cytoagar with 2.25% (w/v) agar (1.800.854.0530, MP Biomedicals) in PBS for 10 min at 4°C. Tubuloids were transferred to an embedding cassette and paraffinized. Subsequently, 5 µm slices were prepared using a paraffin microtome and mounted on FLEX IHC Microscope Slides (Dako, Agilent Technologies). For IHC, tubuloid slices were deparaffinized with 2 × 5 min of incubations in xylene (4055-9010, KLINIPATH) followed by rehydration by dipping in a series of 100%–50% ethanol and finally demi water. Next, target retrieval was performed by boiling in citrate buffer (10 mM sodium citrate, with pH set to 6.0 with citric acid) for 15 min followed by cooling for 30 min at RT and 30 min at 4°C. The slides were then permeabilized for 30 min in TN-buffer (0.15 M sodium chloride, 0.1 M Tris-HCl, pH 7.6) with 0.1% (v/v) Triton X-100 and blocked for 30 min using TN with 0.5% (w/v) blocking reagent from the TSA fluorescein kit (NEL701A001KT, PerkinElmer). The slides with the primary antibodies (Table 3) were incubated overnight at 4°C. Subsequently, slides were washed 3 × 5 min in TN buffer with 0.05% (v/v) Tween 20, followed by incubation with secondary antibodies and DAPI (Table 3). For the AQP3 antibody, TSA amplification was performed as follows. After permeabilization with Triton buffer, endogenous peroxidase activity was blocked with 0.3% (v/v) H_2_O_2_ (23622.298, VWR International) for 30 min and endogenous avidin/biotin binding sites were blocked (Avidin/Biotin Blocking kit (927301, BioLegend)) for 15 min each. After incubation with primary and secondary biotin-conjugated antibodies (Table 3), slices were incubated with streptavidin-HRP for 30 min followed by 7 min incubation with fluorescein tyramide (both TSA Fluorescein System). After mounting with Fluoromount-G (00-4958-02 Thermo Fisher Scientific), images were acquired with laser scanning microscopy (LSM900, Zeiss) objective ×63 (NA 1.4) and processed with FIJI software (ImageJ) (Schindelin et al., 2012). To generate single images of whole tubuloids, multiple images were stitched together (Preibisch et al., 2009).

### ^22^Na uptake experiments

Tubuloids were resuspended in accutase (A6964, Sigma-Aldrich) and incubated for 30 min at 37°C followed by shearing (15x) with a flame-polished pipette until a single cell suspension was reached. Subsequently, 60,000 cells per well were seeded on 24-well cell culture plates (3524, Corning) and grown in EM until confluent. Subsequently, tubuloid monolayers were cultured in CD stimulation medium consisting of BM supplemented with forskolin (10 μM, F6886, Merck), A 83-01 (5 μM, SML0788, Merck) and fludrocortisone acetate (10 μM, F0180000, Merck) for 5 days (Table 1). Before start of the experiment the tubuloids were pre-incubated for 30, 90 or 150 min with uptake buffer (70 mM Na^+^ D-gluconate, 2.5 mM K^+^ D-gluconate, 0.5 mM CaCl_2_, 0.5 mM MgCl_2_ and 2.5 mM HEPES, with pH set to 7.4 using Tris) and inhibitors. The inhibitors used for different conditions were ouabain (1 mM, 102541, MP Biomedicals), hydrochlorothiazide (0.1 mM, H4759, Sigma Aldrich), bumetanide (0.1 mM, B3023, Sigma Aldrich) and/or amiloride (0.1 mM, A7410, Sigma Aldrich) and/or dimethyl sulfoxide (DMSO) as vehicle control. Next, the buffers were replaced with buffers containing tracer ^22^Na (U.S. Department of Energy Isotope Program) for 30 min of incubation at 37°C. Finally, the tubuloids were washed with ice-cold uptake buffer and lysed 15 min with 0.05% (w/v) SDS. The tracer ^22^Na radioactivity was measured using a liquid scintillation counter (Hidex 300SL).

### Statistical analysis

Statistical analysis was performed using One-Way ANOVA combined with Dunnett’s multiple comparisons test or Two-Way ANOVA combined with Sidak or Tukey multiple comparisons test using Prism version 8 (Graphpad, United States). Error bars represent the mean ± standard error of the mean (SEM) and the statistical significance was set at *p < 0.05.

## Results

### Tubuloids can be grown from adult mouse kidneys and differentiated towards CD phenotype

**FIGURE 1:**
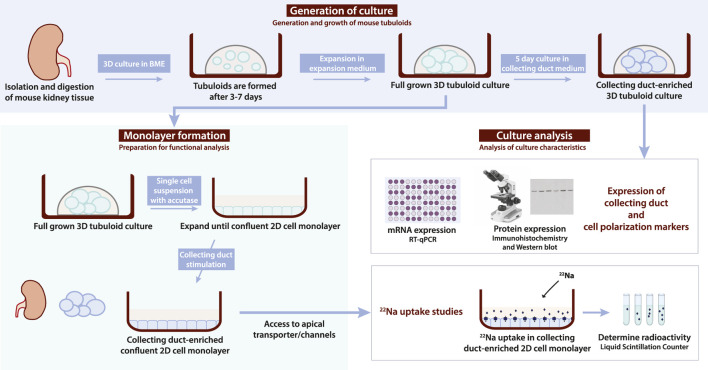
Flowchart depicting mouse tubuloid generation, monolayer formation and analysis protocol. Tubuloids are formed from digested C57BL/6 mouse kidney tissue within 7 days of culture in basement membrane extract (BME) with expansion medium (EM). Subsequent expansion of tubuloids in EM was done to obtain a full-grown 3D tubuloid culture. This full-grown tubuloid culture can be enriched for the CD with 5 days of culture in CD medium. A CD-enriched tubuloid culture can be analyzed with RT-qPCR for mRNA expression or immunohistochemistry and/or Western blot analysis for protein expression to confirm expression of CD (e.g., AQP2 and subunits of ENaC) and/or cell polarization (e.g., ZO-1, E-cadherin, and Na^+^/K^+^-ATPase) markers. Alternatively, a full-grown 3D tubuloid culture can be used for 2D monolayer formation. First, a tubuloid single cell suspension is obtained with accutase treatment from the full-grown 3D tubuloid culture. Subsequently, the single cell suspension is seeded into a normal cell culture plate and grown to a confluent 2D cell monolayer using EM. Once a confluent 2D cell monolayer is formed, it can be stimulated to form a CD-enriched confluent 2D cell monolayer. Growing tubuloids in a 2D cell monolayer allows for access to apical transporters/channels (e.g., ENaC) for functional assays. CD-enriched confluent 2D cell monolayers can be used for functional ^22^Na uptake studies, where radioactive uptake is determined with a liquid scintillation counter. BME; basement membrane extract, EM; expansion medium, 3D; three-dimensional, CD; collecting duct, RT-qPCR; reverse transcription-quantitative PCR, AQP2; aquaporin 2, ENaC; epithelial sodium channel, 2D; two-dimensional.

**FIGURE 2:**
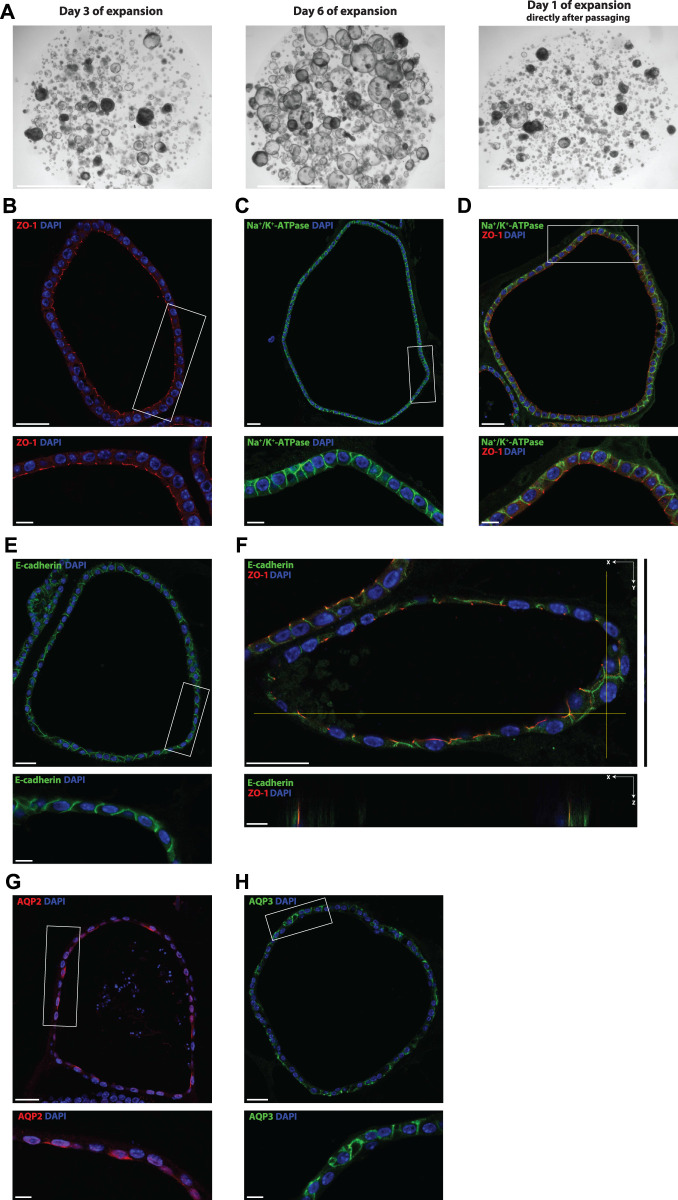
Mouse tubuloids enriched for the CD are polarized and express CD proteins (water channels AQP2/AQP3). Brightfield images of tubuloids growth after 3 and 6 days in culture with EM. Tubuloids were passaged after 7 days in culture, this is referred to as day 1 of expansion directly after passaging **(A)**. Representative immunofluorescence pictures of CD-enriched mouse tubuloids **(B–H)**. Expression of the apical tight junction protein ZO-1 (red) **(B, D, F)**, basolateral Na^+^/K^+^-ATPase (green) **(C, D)** and lateral adherens junction protein E-cadherin (green) **(E, F)**. Staining of CD proteins AQP2, apical water channel, (red) **(G)** and AQP3, basolateral water channel, (green) **(H)** after CD enrichment with CM, which includes forskolin. Inserts (white rectangle) are displayed directly below the main image **(B–H)**. *XZ* direction of Z-stack of the costaining of ZO-1 and E-cadherin is displayed directly below the main image **(F)**. Tubuloids were stained for nuclei (DAPI, blue) **(B–H)**. Scale bars **(A)** 2 mm, **(B–H)** 30 μm and inserts/z-stack 10 μm. CD; collecting duct, EM; expansion medium, CM; collecting duct differentiation medium, ZO-1; zonula occludens 1, Na^+^; sodium, K^+^; potassium, AQP2/3; aquaporin 2/3.

Kidney tubuloids were formed by digesting mouse kidneys from C57BL/6 mice and resuspending digested tissue in basement membrane extract (BME) for subsequent culture (Figure 1). Typically, the first tubuloids developed within 3–7 days of culture in expansion medium (EM) (Table 1) and could be further cultured for at least 10 weekly passages (Figures 1, 2A). With EM, we promoted canonical Wnt signalling, essential for kidney repair (Adams et al., 2010), stimulated proliferation and survival of progenitor cells (Poladia et al., 2006), and inhibited anoikis of dissociated cells (Watanabe et al., 2007).

In this study, we applied the existing human tubuloid culture protocol to the mouse tubuloids and stimulated expression of CD mRNA and proteins (e.g., ENaC and AQP2) (Figure 1). We established a mouse CD-enriched 3D tubuloid culture by a 5-day culture of full-grown tubuloids in CM (Table 1). With CM, we stimulated the enzyme adenylate cyclase, the downstream mediator of AVP (Nielsen et al., 1995), inhibited the mitogen extracellular kinase (MEK1/2), a regulator of cell proliferation and differentiation (Kohno and Pouyssegur, 2006; Degirmenci et al., 2020), and inhibited the TGF-β receptors ALK4/5/7 that promote dedifferentiation and epithelial-to-mesenchymal transition (Tojo et al., 2005). Subsequently, the CD-enriched 3D tubuloid culture was analyzed for expression of CD (e.g., ENaC and AQP2) and cell polarization markers (i.e., ZO-1, E-cadherin and the Na^+^/K^+^-ATPase). Alternatively, the fully grown 3D tubuloid cultures could be formed into a single cell suspension, seeded as 2D cell monolayers, and expanded to a confluent 2D cell monolayer. After stimulation for the collecting duct, the CD-enriched 2D cell monolayers were used for functional ^22^Na uptake experiments (Figure 1).

The morphology of a typical tubuloid culture immediately after passaging, at day 3 and at day 6 is depicted in Figure 2A. CD-enriched tubuloids consisted of a polarized epithelium made up of a single cell layer as shown by expression of the apical zonula occludens 1 (ZO-1), a tight junction-associated protein, (Figures 2B, D, F), basolateral Na^+^/K^+^-ATPase (Figures 2C, D) and the lateral adherens junction protein E-cadherin (Figures 2E, F). Additionally, the *X*Z direction of the ZO-1 and E-cadherin costaining showed a partial overlap of these proteins, which confirms the correct localization of ZO-1, since it acts as a binding partner for both tight and adherens junctions (Campbell et al., 2017). Furthermore, immunofluorescence microscopy showed expression of CD-specific water channels AQP2 and 3 after culture in CM (Figures 2G, H). Specific localization of AQP2 to the apical membrane (Figure 2G) and AQP3 to the basolateral membrane (Figure 2H) further confirmed the polarized CD specificity and response to stimulation with forskolin.

### Collecting-duct enriched mouse kidney tubuloids express water and sodium channels and respond to stimulation with desmopressin

**FIGURE 3:**
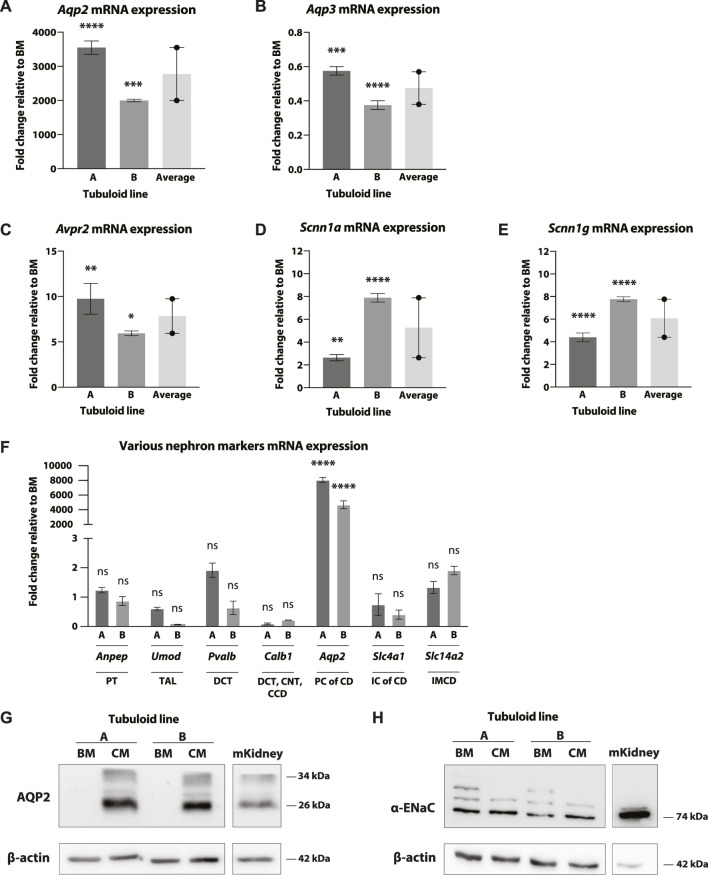
mRNA and protein expression of multiple CD transporters in CD-enriched mouse tubuloids. Fold change mRNA expression of tubuloid line A and B cultured in CD medium (CM) compared to basal medium (BM). mRNA expression of CD water transporters *Aqp2* **(A)** and *Aqp3* **(B)**, the V2R (*Avpr2*) **(C)** and the alpha **(D)** and gamma **(E)** subunits of ENaC (*Scnn1a* and *Scnn1g*) in 2 tubuloid lines, (A, B), after culture in CM (*N* = 2-3 replicates). Average of the 2 tubuloid lines is included in all graphs (*N* = 2 duplicates). mRNA expression of *Anpep* (PT), *Umod* (TAL), *Pvalb* (DCT), *Calb1* (DCT/CNT/CCD), *Aqp2* (PC, CD), *Slc4a1* (IC, CD), and *Slc14a2* (IMCD) in 2 tubuloid lines, (A, B), after CM culture (*N* = 2/4 replicates) **(F)**. Western blot of AQP2 **(G)** and the alpha subunit of ENaC **(H)** in the 2 tubuloid lines after culture with CM and BM. A sample from wild type mouse kidney cortex is included as control. ns, not significant; *p < 0.05, **; *p* < 0.01, ***; *p* < 0.001, ****; *p* < 0.0001. CD, collecting duct; AQP2/3, aquaporin 2/3; V2R, AVP receptor; ENaC, epithelial sodium channel; PT, proximal tubule; TAL, thick ascending limb of the loop of Henle; DCT, distal convoluted tubule; CNT, connecting tubule; CCD, cortical collecting duct; PC, principal cell; CD, collecting duct; IC, intercalating cell; IMCD, inner medullary collecting duct; CM, CD medium; BM, basal medium.

The CD characteristics of mouse kidney tubuloids cultured in CM were further investigated with mRNA and protein expression analysis (Figure 1). Expression levels were compared to culture in simple growth factor withdrawal medium (BM) (Table 1), which, previously, has been shown to increase marker expression of the distal part of the nephron in human tubuloids (Schutgens et al., 2019). Mouse tubuloids differentiated with CM showed a significant increase in mRNA expression of the water channel AQP2 compared to BM for both tubuloid lines assessed (A and B) (Figure 3A). Importantly, tubuloid lines A and B were isolated from 2 different C57BL/6 mice. In addition, Western blot analysis showed clear expression of AQP2 after culturing in CM and complete absence of AQP2 using BM, confirming the CD identity of mouse kidney tubuloids cultured in CM (Figure 3G). Furthermore, mRNA expression analysis showed that the basolateral CD water channel AQP3 could be detected in both tubuloid lines, although expression was somewhat reduced in CM culture (Figure 3B). These quantitative mRNA expression data, together with the basolateral AQP3 staining in immunofluorescence microscopy (Figure 2H) confirm the expression of AQP3 in our culture. Water reabsorption mediated by AQP2 and AQP3 in the CD is promoted by the hormone AVP (Brown, 2003). CD-enriched mouse kidney tubuloids demonstrated clear upregulation of the V2R after culture in CD medium compared to BM in both tubuloid lines (Figure 3C).

Mouse tubuloids differentiated with CM expressed the CD-specific apical Na^+^ channel ENaC, demonstrating a CD enrichment. Quantitative mRNA expression by RT-PCR analysis indicated that both the alpha and gamma subunits of ENaC (*Scnn1a* and *Scnn1g*) were significantly increased in the 2 tubuloid lines after CM culture (Figures 3D, E). Protein expression of the alpha subunit of ENaC was confirmed with Western blot (Figure 3H). We observed relatively high baseline expression of alpha ENaC in BM culture (Figure 3H), compared to the other CD-marker AQP2.

Differentiation with CM led to an increase in markers of the CD and not of other nephron segments in both tubuloid lines. Also, we did not observe an increase in ICs of the CD or in PCs of the inner medullary collecting duct (IMCD). mRNA expression of *Anpep* (Aminopeptidase N, expressed in the PT), *Umod* (Uromodulin, expressed in the thick ascending limb of the LoH (TAL)), *Pvalb* (Parvalbumin, expressed in the DCT), *Calb1* (Calbindin D-28K, expressed in the DCT/CNT/cortical collecting duct (CCD)), *Slc4a1* (Anion exchanger 1, expressed in type A ICs, CD), and *Slc14a2* (Urea transporter A (UT-A), expressed in PCs of the IMCD) was not significantly increased after CM differentiation compared to BM (Figure 3F). Simultaneously, a significant increase in *Aqp2* mRNA expression was observed. These results, together with the absence of expression enrichment of Calbindin D-28K and UT-A, and enrichment of expression of the alpha and gamma subunit of ENaC (Figures 3D, E) suggests that the tubuloid differentiation is directed towards the outer medullary collecting duct (OMCD) rather than to other segments of the CD (CCD or IMCD).

**FIGURE 4:**
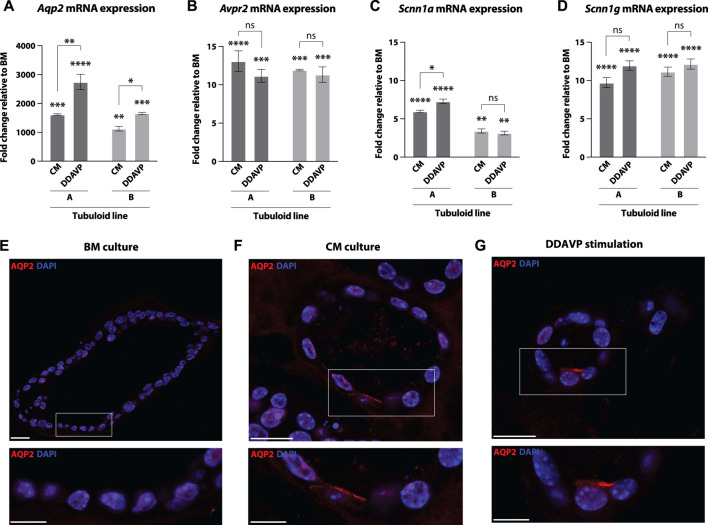
Mouse tubuloids stimulated with DDAVP show increased mRNA expression of various CD transporters and apical expression of the AQP2 protein. Fold change mRNA expression of tubuloid line (A, B) cultured in CD medium (CM) and DDAVP stimulation medium compared to basal medium (BM). mRNA expression of CD water transporter *Aqp2* **(A)**, the V2R (*Avpr2*) **(B)**, the alpha **(C)** and gamma **(D)** subunits of ENaC (*Scnn1a* and *Scnn1g*) in 2 tubuloid lines (A, B), after culture in CM or DDAVP stimulation medium (*N* = 2 duplicates). Representative immunofluorescence pictures of stainings of the apical water channel AQP2 (red) on tubuloids cultured in BM **(E)**, CM (medium including forskolin) **(F)** and DDAVP stimulation medium **(G)**. Inserts (white rectangle) are displayed directly below the main image **(E–G)**. Tubuloids were stained for nuclei (DAPI, blue) **(E–G)**. Scale bars 20 μm and inserts 10 μm. ns, not significant; *p < 0.05, **p < 0.01, ***p < 0.001, ****p < 0.0001. CD, collecting duct; CM, CD medium; DDAVP, desmopressin; BM, basal medium; AQP2, aquaporin 2; V2R, AVP receptor; ENaC, epithelial sodium channel.

Next, the tubuloids were stimulated with DDAVP, a synthetic variant of the hormone vasopressin, to verify that the V2R was physiologically functional in the tubuloid culture (Ufer et al., 1995) (Table 1). We observed a significant increase in mRNA expression of AQP2, the V2R, and the alpha and gamma subunits of ENaC after DDAVP stimulation compared to BM for both tubuloid lines (Figures 4A–D). mRNA expression of AQP2 was significantly increased after DDAVP stimulation compared to CM (Figure 4A), and one of the tubuloid lines also showed a significant increase of the alpha subunit of ENaC upon DDAVP stimulation (Figure 4C). Expression of the V2R and the gamma subunit of ENaC was not changed after DDAVP stimulation compared to CM (Figures 4B, D), thereby showing the similarities between the effect of DDAVP and forskolin (CM medium) (Maric et al., 1998; Hasler et al., 2002). Expression of AQP2 and apical localization after DDAVP stimulation was confirmed with immunofluorescence microscopy. The control BM culture showed an absence of AQP2 (Figure 4E), whereas the CM culture (Figure 4F) and DDAVP stimulation (Figure 4G) induced clear expression and apical localization of AQP2 as expected.

### Mouse tubuloids enriched for the collecting duct show physiological ENaC-mediated Na^+^ uptake

**FIGURE 5:**
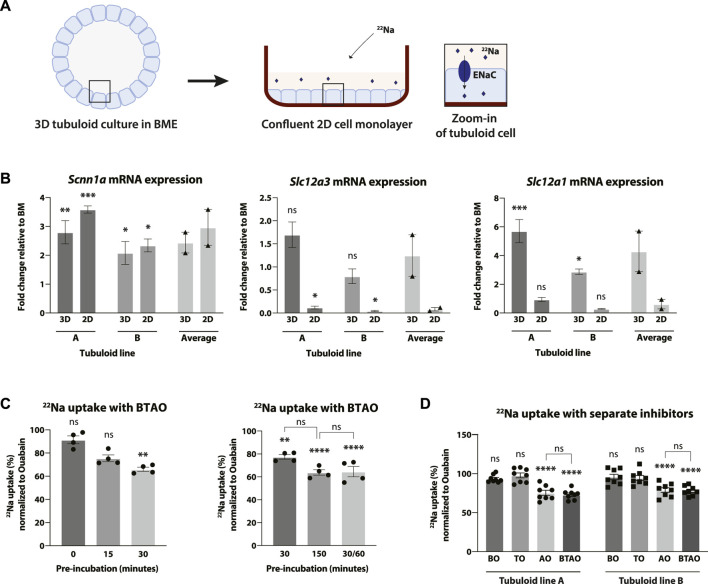
CD-enriched mouse tubuloids show ENaC-mediated uptake of radioactive ^22^Na. Schematic explaining the difference between 3D and 2D in subsequent images of this figure. Tubuloids grown in 3D in a BME gel are formed into a single cell suspension, seeded and expanded into a 2D cell monolayer followed by CD stimulation (see also Figure 1). The 2D cell monolayer allows for apical access of ^22^Na to ENaC for uptake experiments. Apical location of ENaC and entry of ^22^Na in the uptake experiment is schematically shown in the zoom-in of the tubuloid cell **(A)**. Fold change of ENaC (*Scnn1a*), NCC (*Slc12a3*) and NKCC2 (*Slc12a1*) mRNA expression levels in tubuloid lines A and B after 2D and 3D CD stimulation compared to 2D BM culture (*N* = 2 duplicates). Average of the 2 tubuloid lines is included (*N* = 2 duplicates) **(B)**. Uptake of radioactive ^22^Na by tubuloid lines after CD stimulation **(C, D)**. Uptake of ^22^Na in tubuloid line A was tested after pre-incubation periods of 0, 15, 30, and 150 min with 30 min experiment or 30 min pre-incubation with 60 min experiment (30/60). Uptake medium contained multiple inhibitors of transporters; bumetanide (NKCC2), hydrochlorothiazide (NCC) and amiloride (ENaC) (*N* = 4 replicates) **(C)**. The effect of transport inhibitors bumetanide (BO), hydrochlorothiazide (TO) and amiloride (AO), separately, in tubuloid lines (A, B) was compared to the combination of these inhibitors (BTAO) (*N* = 8 replicates from two independent experiments) **(D)**. All experimental conditions include incubation with ouabain (O), an inhibitor of the Na^+^/K^+^-ATPase. Results are normalized to the condition with only ouabain **(C, D)**. ns, not significant; *p < 0.05, **p < 0.01, ***p < 0.001, ****p < 0.0001. 3D, three-dimensional; BME, basement membrane extract; 2D, two-dimensional; ENaC, epithelial sodium channel; NKCC2, Na^+^, K^+^, chloride (Cl^−^) cotransporter; NCC, Na^+^, Cl^−^ cotransporter; CD, collecting duct; BM, basal medium; Na^+^, sodium; K^+^, potassium.

After observing increased CD protein expression in the tubuloids that were differentiated with CM, we investigated whether these proteins were capable of Na^+^ uptake. Because mouse tubuloids in BME consist of polarized cell layers with an enclosed apical compartment facing the lumen (Figures 2B, D, F), we developed a tubuloid cell monolayer culture to gain access to the apical membrane for functional studies (Figures 1, 5A). Tubuloids were formed into a single cell suspension, seeded and grown into a confluent 2D cell monolayer, and stimulated to generate CD-enriched confluent 2D cell monolayers with access to apical transporters/channels. Here, ENaC function and activity was further stimulated with fludrocortisone (Table 1).

To determine the effect of CD simulation with fludrocortisone in 2D and 3D tubuloids (Figure 5A), the expression of several markers of the distal parts of the nephron including α-ENaC (*Scnn1a*), NCC (*Slc12a3*, expressed in the DCT) and NKCC2 (*Slc12a1*, expressed in the TAL) was verified with reverse transcription-quantitative PCR. mRNA expression of the alpha subunit of ENaC was significantly increased similarly in both 2D and 3D CD stimulation compared to BM in both tubuloid lines. mRNA expression of NCC did not change significantly with CD stimulation in 3D, but was significantly downregulated in 2D. NKCC2 expression was significantly increased upon CD stimulation in 3D, but not in 2D (Figure 5B). Following these results, we concluded that 2D monolayer culture to access the apical compartment was also suitable for CD enrichment and, thus, for functional studies.

Next, functional uptake of the radioactive isotope ^22^Na by the CD-enriched tubuloid monolayers was studied. The tubuloid monolayers were capable of ^22^Na absorption as shown by the uptake of the radioactive ^22^Na and the subsequent reduction in uptake after inhibition of the main Na^+^ transporters of the distal part of the nephron (Figures 5C, D). The channels/transporters ENaC, NCC and NKCC2 were inhibited using amiloride (A), hydrochlorothiazide (T) and bumetanide (B), respectively. In addition, ouabain (O) was added in all conditions to prevent basolateral extrusion of ^22^Na by the Na^+^/K^+^-ATPase. The ^22^Na uptake experiments included a pre-incubation with uptake buffer including the inhibitors before the start of the experiment to allow the inhibitors to take effect. First, the optimal pre-incubation time was determined by performing ^22^Na uptake with the cocktail of Na^+^ transporter/channel inhibitors (BTAO). The decrease in uptake of ^22^Na in response to apical inhibitors was significant after 30 and 150 min of pre-incubation, but not for 0 and 15 min (Figure 5C). There was no significant difference between 30 and 150 min of pre-incubation, but there was a trend suggesting that 150 min might lead to a greater effect of BTAO. Also, a pre-incubation of 30 min followed by 60 min of experiment time (30/60) showed a similar, non-significant decrease in uptake compared to the 150 min of pre-incubation (Figure 5C). Therefore, we concluded that the optimal pre-incubation time for the inhibitors was 150 min.

To determine which apical channel(s) and/or transporter(s) were responsible for the ^22^Na uptake, the individual effects of the inhibitors were determined (Figure 5D). As seen in previous experiments (Figure 5C), CD-enriched mouse tubuloid monolayers showed physiological uptake of ^22^Na which was significantly inhibited by BTAO. In addition, the individual effects of the inhibitors clearly showed that this uptake was directly mediated by the amiloride-sensitive ENaC as ^22^Na uptake was significantly reduced after inhibition of ENaC with amiloride, with a similar, non-significant reduction compared to the condition with all inhibitors (BTAO). This demonstrates that the ^22^Na uptake in the tubuloids was significantly mediated by ENaC. We were not able to detect a significant reduction in ^22^Na uptake by inhibition of NKCC2 and NCC alone, which is in line with expression data of these electrolyte transporters.

## Discussion

Our current knowledge of kidney (patho)physiology is mainly derived from conventional research models that remain insufficient to fully elucidate certain mechanisms of kidney (patho)physiology (Jung and Kwon, 2016; Edwards and Crambert, 2017; Kleyman and Eaton, 2019). And even though many models have been described, they often lack endogenous expression of relevant ion channels and transporters that are subject to physiological regulation (Yang et al., 2022). Therefore, we have created tubuloids derived from mouse kidney tissue that are differentiated towards the CD, show physiological channel regulation and functional ion uptake. Previous studies have shown that human tubuloids can be stimulated to increasingly express physiological relevant proteins of certain nephron segment(s) in response to (bio)mechanical and (bio)chemical cues or the lack thereof (e.g., growth factor withdrawal) (Schutgens et al., 2019). We have utilized this plasticity of tubuloids and developed a CD medium that resulted in a remarkable increase of CD proteins that are essential for kidney function (i.e., AQP2 and ENaC), thereby enriching this mouse tubuloid culture for the CD. More specifically, the upregulation of ENaC combined with a lack of enrichment of calbindin D-28K, expressed in the CCD, and UT-A, expressed in the IMCD, suggests that our culture most likely represents the OMCD (Chen et al., 2021). Further characterization of this tubuloid OMCD representation is required to determine protein expression and localization. The upregulation of ENaC in the mouse tubuloid culture on protein level was modest compared to AQP2, which might be explained by the presence of insulin in the medium (a component of advanced DMEM/F12), which is known to indirectly stimulate ENaC expression (Tiwari et al., 2007). We did observe a reduction in the number of additional bands on the alpha ENaC Western blot upon CD-enrichment. This might suggest increased maturity of ENaC since the mouse kidney control showed a similar profile. Also, the presence of alpha ENaC fragments >70–80 kDa in mouse tissue has been shown before by multiple groups (Zhang et al., 2016; Frindt et al., 2020; Bohnert et al., 2021; Artunc et al., 2022). The translocation of AQP2 to the apical membrane of the tubuloids indicates a physiological response to the CD medium component forskolin, which mimics the effect of AVP by raising intracellular cyclic adenosine monophosphate (cAMP) levels (Seamon et al., 1981; Rice et al., 2012). This activates protein kinase A (PKA) which phosphorylates AQP2, thereby triggering its trafficking to the apical membrane (Olesen and Fenton, 2017). Tubuloids also showed a physiological response to stimulation with DDAVP, a synthetic variant of AVP, by upregulation of CD markers and translocation of AQP2 to the apical membrane. This confirms the presence of a functional V2R and associated signalling pathways in mouse tubuloids. For functional studies, ENaC expression and activity was further stimulated by fludrocortisone, an aldosterone analogue and mineralocorticoid agonist (Yamamoto et al., 2016). The CD-enriched mouse tubuloids were capable of amiloride sensitive Na^+^ uptake, demonstrating ENaC-specific Na^+^ transport. Therefore, the presented mouse tubuloids allow to easily isolate epithelial cells that are expandable while maintaining a physiological expression profile and function.

A major advantage of mouse tubuloids is that they can be easily derived from existing (diseased) mouse models for complementary *in vitro* studies while simultaneously reducing the need for additional animal experiments. Our CD differentiation protocol with innate tubuloid AQP2 and ENaC expression and function is of added value for future studies of CD function and regulation. This also includes pathogenic phenotypes (e.g., NDI and Liddle syndrome) that arise from this segment. For example, tubuloids can be obtained from existing mouse (knockout or mutation) models of the V2R, AQP2, and ENaC, thereby providing a model for e.g. hereditary NDI and Liddle’s syndrome. Although the molecular mechanisms of Liddle syndrome are well-studied and amiloride is used as an effective therapy against the significant hypertension that many patients suffer from, it has been found that not all patients respond the same way to this therapy which leaves them susceptible for hypertension-induced risks (Pradervand et al., 1999). NDI is a more prevalent disease and novel mutations have been discovered recently (Gao et al., 2020; Li et al., 2021). However, these studies suggest that some mechanisms of, e.g., AQP2 trafficking remain to be unveiled (Olesen and Fenton, 2021). Because these diseases have low prevalence, patient sample size and lack of clinical data are limitations to further unveil the pathophysiology and improved (personalized) treatments (Enslow et al., 2019; Fan et al., 2020). Here, tubuloids from existing mouse models can be of added value to study these tubulopathies.

Limitations of this study include the heterogeneity of the tubuloid culture and the lack of IC enrichment. Although the CD medium significantly upregulates CD specific proteins, other segments remain present in the culture. Whereas this can be considered as an advantage over single cell type cultures, it can be a disadvantage for some applications. For example, our uptake experiment did not completely diminish the Na^+^ uptake after ENaC inhibition with amiloride. This indicates that the CD-enriched tubuloid culture might still express Na^+^ transporters of other nephron segments. As such, human tubuloids have been shown to express multiple proximal tubule markers, suggesting a large presence of this segment in the culture (Schutgens et al., 2019). For applications that require a homogenic CD culture, cell sorting methods and/or creation of CD reporter lines should be investigated. Examples include growing tubuloids from existing mouse strains with fluorescent-labeled reporter lines for more convenient sorting (Zharkikh et al., 2002; Miller et al., 2006). In addition to the heterogeneity of the culture, we also observed differences in the magnitude of effect between the two tubuloid lines. This suggests that interindividual differences might also play a role in the maturity/functionality. Future studies should further explore the importance of this effect in the CD-enriched tubuloid culture. Furthermore, the CD differentiation medium does not seem to increase expression of ICs. Similar to our results, Shi et al. (2022) recently showed an absence of ICs in their original organoid culture, which could be restored by overexpression of the transcription factor FOXI1. For applications that require ICs, the expression of ICs in the CD-enriched tubuloid culture should be further investigated and the culture procedure might be adapted to increase expression of FOXI1 or other IC transcription factors.

We believe that our CD-enriched tubuloid culture is a unique tool to study CD (patho)physiology. Tubuloids allow for easy accessibility for functional studies, are versatile and easily usable. Our culture system can be used complimentary to iPSC-derived organoids that mimic nephrogenesis. Although these organoids include multiple cell types including stromal and vascular cells, they lack a mature expression profile (Wu et al., 2018). Presence of these cell types increases similarities with the *in vivo* situation, but it also increases complexity for certain experiments. For example, the access to certain cells or specifically the apical compartment is more difficult and the multiple cell types that are present can interfere with the readout. Recent studies have shown efforts to obtain specifically kidney epithelial cells from organoids by either more directed differentiation or isolation of epithelial cells (Montalbetti et al., 2022; Shi et al., 2022). These approaches have enabled functional characterization, including amiloride-sensitive ENaC activity specific to the collecting duct, which would have not been feasible with original organoid protocols. These are valuable developments in the field that allow for functional studies. In this regard, the purely epithelial nature of tubuloids allow for different studies of specific effects on the epithelial cells alone without the interference of other cell types.

In addition to the (bio)chemical cues that can stimulate the expression of certain markers in the tubuloid culture, mechanical cues are also essential. A tubuloid-on-a-chip has been described by Gijzen et al. (2021) which kept the 3D environment of the tubuloids while allowing access to both apical and basolateral sides and applying shear stress. Since the *in vivo* tubular lumen is subjected to shear stress due to the flow of pre-urine, enabling flow *in vitro* will allow for a more physiological experimental set-up. This is also the case specifically for AQP2 and ENaC, as both channels have been known to be regulated by (bio)mechanical cues including shear stress (Jang et al., 2011; Shi et al., 2013; Cosgun et al., 2022). In this regard, incorporating mouse tubuloids into an organ-on-a-chip system can increase the translational value in future studies.

In conclusion, this study presents a new mouse kidney tubuloid model that can be differentiated towards the CD. CD-enriched mouse tubuloids form a polarized epithelium in 3D that consists of a single epithelial cell layer. This tubuloid culture expresses key channels and transporters of the CD in a polarized fashion and demonstrates CD-specific channel regulation and electrolyte uptake. Therefore, mouse kidney tubuloids allow for future studies of CD physiology and may help to further improve our knowledge of kidney physiology and pathophysiology of (rare) diseases.

## Data Availability

The raw data supporting the conclusion of this article will be made available by the authors, without undue reservation.
